# Assessment of an instrument scale measuring the knowledge of antiretroviral therapy among people living with HIV

**DOI:** 10.1186/s12889-023-15220-x

**Published:** 2023-02-07

**Authors:** Di Xu, Yuhua Shi, Ling Pan, Qiongli Duan, Nengmei Huang, Pengcheng Liu, Jing Han, Zhongfu Liu, Jian Li, Hongjie Liu

**Affiliations:** 1 National Center for AIDS/STD Control and Prevention, Chinese Center for Disease Control and Prevention, 155 Changbai Road, Changping District, 102206 Beijing, People’s Republic of China; 2 Shanghai Municipal Center for Disease Control & Prevention, Shanghai, People’s Republic of China; 3 Yunnan Center for Disease Control and Prevention, Kunming, People’s Republic of China; 4 Center for Disease Control and Prevention in Honghe Hani and Yi Autonomous Prefecture, Mengzi, People’s Republic of China; 5 Department of Epidemiology and Biostatistics, School of Public Health, University of Maryland, College Park, MD USA

**Keywords:** Psychometric assessment, Instrument scale, Knowledge of antiretroviral therapy

## Abstract

**Background:**

Antiretroviral therapy (ART) is currently the most effective way to treat people living with human immunodeficiency virus (PLHs) and reduce HIV transmission. While there are many factors that reduce adherence to ART, PLHs’ knowledge about ART may determine the level of adherence. It is necessary to design and assess an instrument scale that measures the knowledge of antiretroviral therapy among PLHs.

**Method:**

A cross-sectional study was conducted among PLHs in Honghe Hani and Yi Autonomous Prefecture, China. Both exploratory and confirmatory factor analyses were used to examine the latent factors of antiretroviral therapy knowledge scale. Internal consistency was assessed separately for the scale and its dimensions by estimating Cronbach’s alphas, split-half reliability and Spearman’s correlation coefficient. ANOVAs were used to compare the scores of different dimensions with sociodemographic characteristics.

**Results:**

Four factors were extracted according to factor loadings. They had high internal consistency reliability (Cronbach’s alpha: 0.70–0.95) and good construct validity (standardized factor loading range: 0.46–0.86) in the scale. Goodness of fit indices indicated that a four-factor solution fit the data at an accepted level (χ^2^/degree ratio = 1.980, RMSEA = 0.069, GFI = 0.909, CFI = 0.957, NFI = 0.917, TLI = 0.944). ANOVAs indicated that the score was higher among PLHs who were Han, had spouses/partners, were non-famers or migrant workers, initiated ART, and had a high school or above education.

**Conclusion:**

The psychometric assessment indicated that this ART knowledge scale had accepted internal consistency and discriminant construct validity. It can be used to assess the knowledge of antiretroviral therapy for PLHs.

## Background

The human immunodeficiency virus (HIV)/acquired immune deficiency syndrome (AIDS) remains worldwide one of the major infectious diseases that impair public health. Globally, 37.7 million people were living with HIV (PLHs) by the end of 2020 [1]. Reducing mortality and improving quality of life in the context of PLHs has been one of the major objectives of the HIV/AIDS control and prevention. Antiretroviral therapy (ART) is currently the most effective measure to treat PLHs and reduce HIV transmission [2–4].

To control the HIV epidemics, UNAIDS established the goal of the 90–90-90 in 2014. The goals called for 90% of all PLHs knew their HIV status, 90% of all people with diagnosed HIV infection received sustained antiretroviral therapy, and 90% of all people receiving antiretroviral therapy had viral suppression by 2020 [5]. However, this goal has not been achieved. Only 84% (67–98%) knew their status, 73% (57–88%) were accessing ART, and 66% (53–79%) were virally suppressed [1]. Later, UNAIDS proposed the “4 95% Targets” of the Global AIDS Strategy 2021–2026 [6]. The targets called for 95% of people at risk of HIV infection use combination prevention, 95% of PLHs know their HIV status, 95% of PLHs know their status initiate treatment and 95% on treatment are virally suppressed [6]. Achieving these goals is difficult with challenges. One of the challenges is adherence to ART as this therapy will be used lifelong. While there are many factors that reduce adherence to ART, PLHs’ knowledge about ART may determine the level of adherence [7, 8]. As previously reported, high knowledge of ART is associated with access, use and adherence to ART, [8] while a lack of knowledge about ART leads to reduced adherence and incorrect use of drugs [9].

To effectively increase ART initiation and adherence for PLHs, it is necessary to develop intervention programs that, as the initial step, increase PLHs’ knowledge of ART. To date, few measurement scales have been designed to measure the level of ART knowledge for developing countries [8]. To fill the gap, we designed and assessed a measurement scale that can be used in measuring ART knowledge among PLHs.

## Materials and methods

### Study sites and participants

In 2021, we conducted a cross-sectional study among PLHs in Honghe Hani and Yi Autonomous Prefecture (Honghe Prefecture), Yunnan province, where the HIV prevalence was high [10]. Honghe Prefecture is located in the southwest of China, near Laos and Vietnam. Since the first reported HIV case of drug user in 1995, the number of reported cases in Honghe Prefecture has been increasing. At the end of 2018, the estimated overall HIV prevalence was 89/100,000 (1.25 million/1.4 billion) in China and 765/100,000 (35,977/4.7 million) in Honghe Prefecture, making it one of the most HIV-affecting areas in China [11, 12]. HIV transmission mode in Honghe Prefecture has evolved from drug use transmission mode to a predominantly sexual transmission mode in recent years, with the proportion of sexual transmission reaching 85–93% [13].

Participants were recruited by using a convenience sampling method. Generally, PLHs were recommended to be followed up per 6 months by local CDCs and per 3 months by designated ART clinics for those prescribed ART in China. The potential participants who visited the local CDCs or designated ART clinics were first asked their willingness to participate in the study. PLHs who were eligible for participation and provided informed consents were interviewed by trained local healthcare providers in a private interview room. Subjects were eligible if they were 18–69 years old, infected with HIV by the heterosexual transmission mode, and provided informed consent. Potential participants where were excluded if they had severe mental illness, mental deficiency, and language barrier. The study protocol was approved by the Ethics Committee of the National Center for AIDS/STD Control and Prevention, Chinese Center for Disease Control and Prevention.

### Measurements

To develop the scale of ART knowledge, we conducted a literature review of existing questionnaires of HIV and ART related knowledge. The knowledge scale was intended to cover knowledge about impact of ART on HIV progression, consequences of non-adherence, side effects, and benefits of successive treatment [14, 15]. After extensive literature review, we designed a scale that measured four facets of ART, including the awareness of ART medication, drug adherence, treatment benefits, and adverse effects. The drafted items were discussed and revised by our research team. Emphasis was put on the applicability to the Chinese context.

The 14-item scale consisted of core domains for knowledge regarding the ART. There were three options for each item, including “right”, “wrong” and “unknow”. One point was given for each correct answer and no point for incorrect or unknown answers, with a total score ranging from 0 to 14 points. Score was dichotomized into low (0–8) and high (9–14) on the basis of its distribution. High score indicated better understanding of ART. Sociodemographic characteristics were also collected in the questionnaire. The questionnaire was piloted among 20 PLHs in study sites. Questions were revised and modified according the findings from the pilot, with the aim of making the items more understandable to the participants.

### Data analysis

Psychometric property assessment was performed in internal consistency analysis (Cronbach’s alphas, split-half reliability and Spearman’s correlation), construct validity (factor analysis) and computation of scale scores generally [16].

Exploratory and confirmatory factor analysis were used to assess the construct validity. The total sample were divided randomly into two independent samples. In sample one, exploratory factor analysis with oblique rotation was conducted to assess the dimensionality of the scale that underpinned the sample data [16]. Factor analysis was conducted on selected items, using the principal-components factor-extraction method to ascertain factors and factor loadings [17, 18]. Items with an absolute value loading of 0.40 or greater were retained [19]. Cumulative proportions of variance were obtained from continuous factor solutions.

In sample two, confirmatory factor analysis was conducted to verify the factor structure obtained from exploratory factor analysis. Goodness of fit was assessed using chi-square test of exact fit (non-significant *p*-value as a good fit), root mean square errors of approximation (RMSEA; ≤0.05 as a good fit and ≤ 0.07 as an acceptable fit), goodness of fit index (GFI; ≥0.90), comparative fit index (CFI; ≥0.90) and Tucker Lewis index (TLI; ≥0.90) [20]. As each fit index had its own strengths and weaknesses, various types of fit indices in tandem with the particular aspects were also considered in determining the model fit [21]. For example, when the RMSEA closed to 0.07 may be of less concern if all other indices were strongly in a range indicating good model fit. A composite score was also calculated for each of the scale and the dimensions. Descriptive statistics, including mean and standard deviation (SD) values for each score distribution, were presented for the scale. ANOVAs were used to compare the scores of different dimensions with sociodemographic characteristics.

Internal consistency assessment was conducted separately for the scale and each dimension by calculating Cronbach’s alphas and split-half reliability [22]. A scale with an alpha of ≥0.7 was accounted as have good to excellent reliability [23]. Spearman’s correlation coefficient was calculated among each factor, *P* < 0.05 indicates good consistency [23]. Data were analyzed by IBM SPSS ver. 24.0 (IBM Corp., Armonk, NY) and R 4.1 software (lavaan package).

## Results

### Characteristics of participants

A total of 410 eligible PLHs completed the questionnaire during the study period. The mean age of participants was 47.37 years (SD = 12.04). The majority of the PLHs were male (64.7%), had primary school education or junior high school education levels (86.2%), were farmers or migrant workers (61.6%), and were without spouses/partners (52.6%) at the time of interview (Table 1).

**Table 1:** Sociodemographic and infection characteristics of the participants in Honghe Prefecture in 2021

Characteristics	N	Proportion (%)
Gender		
Male	266	64.9
Female	144	35.1
Age/years		
19~	71	17.3
36~	113	27.6
46~	96	23.4
56~	130	31.7
Ethnicity		
Han	203	49.5
Others	207	50.5
Education level		
≤Primary school	215	52.4
Junior high school	139	33.9
≥Senior secondary school	56	13.7
Marital Status		
With spouses/partners	193	47.1
Without spouses/partners	217	52.9
Occupation		
Farmers or migrant workers	252	61.5
Others	158	38.5
CD4 counts (/μL)		
≤200	64	15.6
201 ~ 350	122	29.7
351 ~ 500	104	25.4
501~	89	21.7
Not detected	31	7.6
ART Status		
No	145	35.4
Yes	265	64.6

### Exploratory factor analysis

**Fig. 1:**
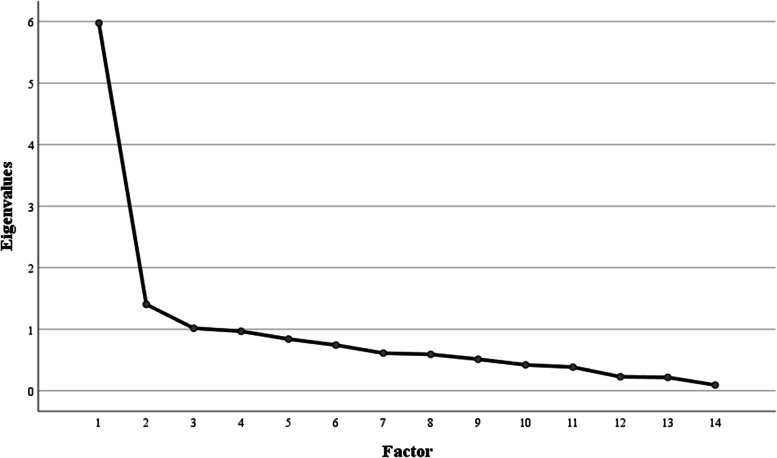
Scree test for Eigenvalues in the development sample

The findings of exploratory factor analysis suggested a four-factor response. Using eigenvalue > 1 criterion [24], four factors which had eigenvalues > 1 were retained in the development sample. As showed in the scree plot (Fig. 1), eigenvalues of the first four factors were > 1 and the curve flattened out from factor 4, suggesting that factor 1, 2, 3 and 4 should be retained. Table 2 presented items and their loadings for four factors. An item was considered to load on a targeted factor if the item had a loading factor that was ≥0.4 for that factor and was < 0.4 for all others [25]. A total of seven items loaded on the first factor, which measured the awareness of ART. Two items loaded on factor 2 and factor 3, which assessed the understanding of treatment benefits and drug adherence, separately. The remaining three items loaded on the fourth factor, which was knowledge of adverse effects. Table 3 conveyed eigenvalues and variance by factors. The eigenvalues of the four factors counted 66.87% of the total variance: 18.94% for factor 1-knowledge of ART medication, 16.40% for factor 2-knowledge of treatment benefits, 16.18% for factor 3-knowledge of drug adherence and 15.35% for factor 4-knowledge of adverse effects.

**Table 2:** Items and corresponding factor loadings from the rotated factor structure matrix: principal axis factoring with a Varimax rotation

Item	Factor 1	Factor 2	Factor 3	Factor 4
*q1. No ART is needed absence of symptoms.*	0.46*	0.36	0.20	0.30
*q2. Early initiation of ART can improve outcomes for PLHs.*	0.55*	0.28	0.26	0.11
*q3. Stop taking drugs once you feel better.*	0.57*	0.28	0.38	0.23
*q4. ART requires lifelong daily oral therapy.*	0.83*	0.07	0.20	0.27
*q5. Drugs can be stopped with undetectable viral load.*	0.80*	0.12	0.15	0.22
*q6. Condoms can be avoided with undetectable viral load.*	0.60*	0.33	0.00	0.14
*q7. Drug resistance will develop with irregular medication.*	0.25	0.13	0.84*	0.29
*q8. Drug resistance will develop with omitted dose.*	0.17	0.18	0.85*	0.32
*q9. Drugs have certain side effects.*	0.22	0.03	0.12	0.75*
*q10. It can be relieved by reducing dose when side effects occur.*	0.28	0.09	0.14	0.72*
*q11. Side effects of drugs can be relieved or disappear after 2 to 6 weeks.*	0.11	0.24	0.23	0.75*
*q12. ART prevents sexual transmission of HIV.*	0.09	0.86*	0.21	0.16
*q13. ART prevents mother-to-child transmission of HIV.*	0.13	0.85*	0.24	0.03
*q14. ART prolongs lives of PLHs until he/she resembles normal.*	0.56*	0.22	0.34	−0.01

**Table 3:** Eigenvalues and variance explained by factors

Factors	Eigenvalues	% Variance	% Cumulative
1	5.98	18.94	18.94
2	1.40	16.40	35.34
3	1.02	16.18	51.52
4	1.00	15.35	66.87

### Confirmatory factor analysis

**Fig. 2:**
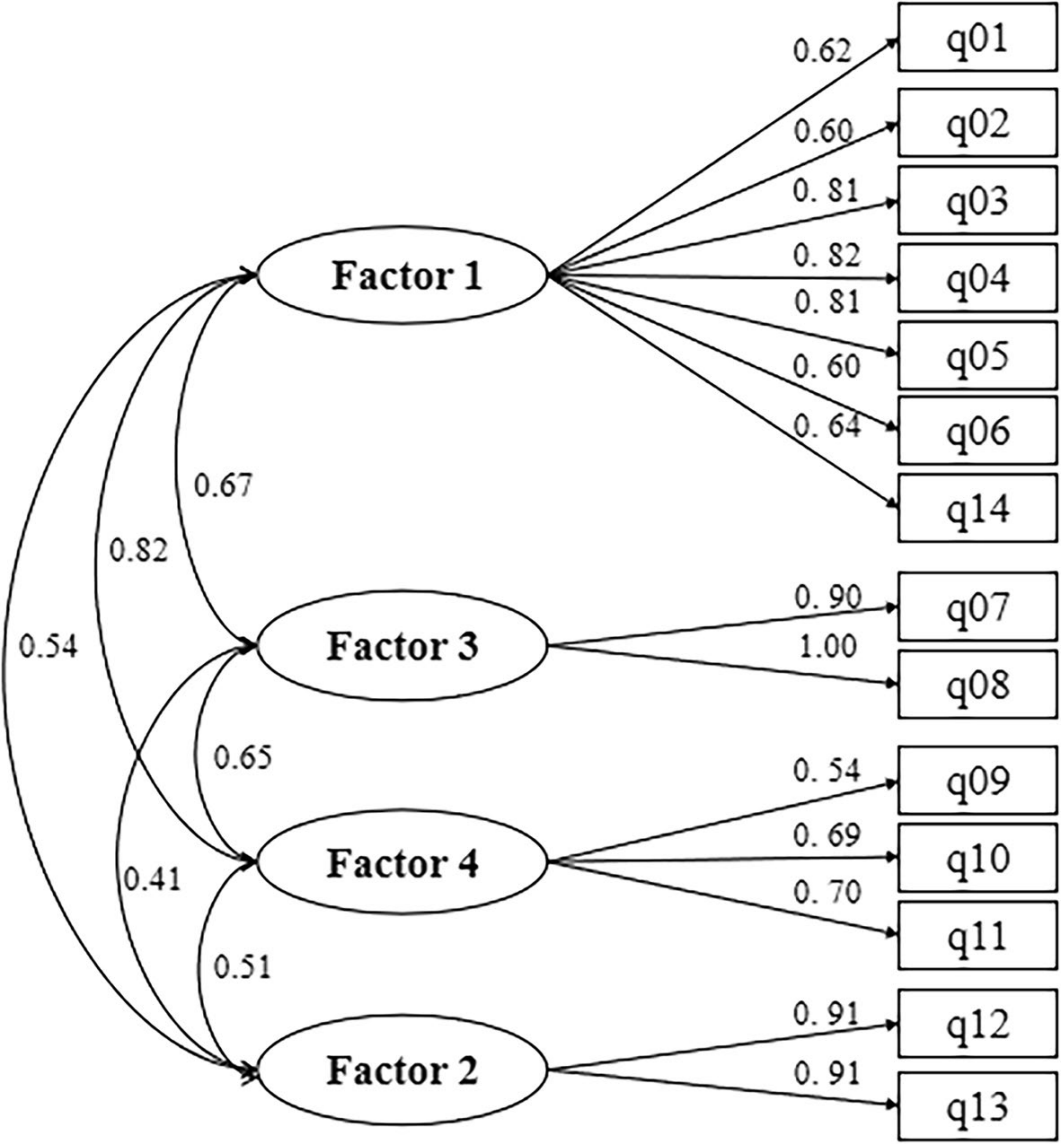
Standard path and parameter estimation of confirmatory factor analysis. Factor 1, the cognition of ART medication; Factor 2, the cognition of Treatment benefits; Factor 3, the cognition of Drug adherence; Factor 4, the cognition of Adverse effects

To determine the optimal dimension structure of the scale, basing on the R Studio, the researcher used the maximum likelihood method to carry out the confirmatory factor analysis of the 14-item and 4-factor structure of ART scale. The correlation coefficients among the four dimensions were 0.67, 0.82, 0.54, 0.65, 0.41 and 0.51. The standardized path coefficients between the dimensions and the subscales ranged from 0.54 to 0.91. The path coefficients of most items over 0.50 indicated that ART scale has a large effect with great path association. The results indicated that a four-factor solution fit the data at an acceptable level in the verification sample (goodness of fit indices as χ^2^/degree ratio = 1.980, RMSEA = 0.069, GFI = 0.909, CFI = 0.957, NFI = 0.917, TLI = 0.944). The standard path and parameter estimation of confirmatory factor analysis is shown in the Fig. 2.

### Reliability

The detection of Cronbach’s alphas and split-half reliability were 0.901 and 0.875 respectively. All the Cronbach’s alphas and split-half reliability of four dimensions were acceptable, all of which were higher than 0.7 except for the understanding of adverse effects (0.68). There were no substantial differences on the estimated alpha after deleting each item, indicating the strong correlation between each item and the scale. Every item was valid and credible enough to satisfy the requirements of statistical analysis. The results showed the 4 sub-scales had strong reliability (Table 4).

**Table 4:** Cronbach’s coefficient alphas and correlation of four factors in the scale

				Cronbach’s coefficient alphas		Correlation		
Factors	Items^*a*^	Means^*b*^	SD^*c*^	Whole sample	split-half reliability	1^*d*^	2^*e*^	3^*f*^
ART medication	7	4.86	2.29	0.85	0.84	1.00		
Drug adherence	2	1.31	0.93	0.95	0.95	0.60(*P* < 0.01)	1.00	
Adverse effects	3	1.89	1.12	0.70	0.68	0.62(*P* < 0.01)	0.53(*P* < 0.01)	1.00
Treatment benefits	2	1.05	0.95	0.88	0.88	0.52(*P* < 0.01)	0.39(*P* < 0.01)	0.35(*P* < 0.01)

^a^ items of each dimension;

^b^ range: 2–7;

^c^ standard deviation;

^d^ ART medication;

^e^ Drug adherence;

^f^ Adverse effects

ANOVAs were used to compare the divergence among different characteristics. The score of each dimension was the sum of all entries of that dimension and the total score was the sum of each item. The scores were higher among PLHs who were Han, had spouses/partners, were non-famers or migrant workers, received ART and had junior high school or above education levels respectively (Table 5).

**Table 5:** Each dimension’s and the scale’s score among different characteristics of participants in Honghe Prefecture in 2021

Characteristics	ART medication	Drug adherence	Adverse effects	Treatment benefits	Overall score
Gender					
Male	4.82 ± 2.32	1.26 ± 0.94	1.83 ± 1.13	1.08 ± 0.94	8.99 ± 4.41
Female	4.92 ± 2.27	1.41 ± 0.89	1.99 ± 1.09	1.00 ± 0.97	9.31 ± 4.18
*F value*	0.158	2.471	1.827	0.657	0.517
*P value*	0.691	0.117	0.177	0.418	0.473
Age/years					
19~	4.25 ± 2.62	1.13 ± 0.96	1.58 ± 1.14	1.04 ± 0.97	8.00 ± 4.96
36~	5.05 ± 2.40	1.34 ± 0.92	1.96 ± 1.12	1.09 ± 0.95	9.39 ± 4.38
46~	4.95 ± 2.04	1.39 ± 0.90	1.92 ± 1.09	1.03 ± 0.96	9.28 ± 3.85
56~	4.94 ± 2.16	1.32 ± 0.93	1.91 ± 1.12	1.04 ± 0.93	9.31 ± 4.20
*F value*	2.065	1.221	2.175	0.007	1.895
*P value*	0.104	0.302	0.090	0.972	0.130
Ethnicity					
Han	5.22 ± 1.97	1.47 ± 0.86	2.08 ± 1.05	1.14 ± 0.93	9.91 ± 3.75
Others	4.50 ± 2.54	1.15 ± 0.97	1.69 ± 1.15	0.97 ± 0.96	8.31 ± 4.71
*F value*	10.407	12.023	13.021	3.399	14.500
*P value*	0.001*	0.001*	< 0.001*	0.066	< 0.001*
Education level					
≤Primary school	4.47 ± 2.50	1.16 ± 0.96	1.67 ± 1.14	0.93 ± 0.95	8.23 ± 4.46
Junior high school	5.29 ± 2.01	1.53 ± 0.84	2.06 ± 1.09	1.20 ± 0.92	10.07 ± 3.90
≥Senior secondary school	5.25 ± 1.94	1.35 ± 0.92	2.28 ± 0.90	1.18 ± 0.30	10.05 ± 3.31
*F value*	8.825	3.155	9.740	1.581	9.612
*P value*	< 0.001*	0.001*	< 0.001*	0.207	< 0.001*
Marital Status					
With spouses/partners	5.16 ± 2.12	1.42 ± 0.89	2.11 ± 1.06	1.16 ± 0.94	9.86 ± 3.99
Without spouses/partners	4.58 ± 2.42	1.21 ± 0.95	1.69 ± 1.13	0.95 ± 0.94	8.43 ± 4.52
*F value*	6.743	5.023	15.129	5.381	11.431
*P value*	0.010*	0.026*	< 0.001*	0.021*	0.001*
Occupation					
Farmers or migrant workers	4.67 ± 2.44	1.21 ± 0.95	1.74 ± 1.14	1.04 ± 0.94	8.67 ± 4.61
Others	5.15 ± 2.03	1.47 ± 0.87	2.11 ± 1.04	1.06 ± 0.96	9.79 ± 3.75
*F value*	4.109	7.642	10.998	0.042	6.594
*P value*	0.043*	0.006*	0.001*	0.838	0.011*
CD4 counts (/μL)					
≤200	2.42 ± 2.23	0.55 ± 0.85	0.55 ± 0.81	0.68 ± 0.91	4.19 ± 1.04
201 ~ 350	4.89 ± 2.28	1.44 ± 0.90	1.92 ± 1.09	0.91 ± 0.92	9.19 ± 4.34
351 ~ 500	4.78 ± 2.49	1.24 ± 0.95	1.78 ± 1.16	1.00 ± 0.97	8.80 ± 4.57
501~	5.13 ± 2.05	1.42 ± 0.90	2.07 ± 1.05	1.18 ± 0.96	9.81 ± 3.97
*F value*	11.798	6.993	16.775	2.480	14.303
*P value*	< 0.001*	< 0.001*	< 0.001*	< 0.044*	< 0.001*
ART Status					
No	3.01 ± 2.45	0.81 ± 0.95	1.01 ± 0.99	0.70 ± 0.89	5.54 ± 4.54
Yes	5.86 ± 1.42	1.58 ± 0.79	2.36 ± 0.0.87	1.25 ± 0.92	11.05 ± 2.64
*F value*	222.047	75.940	203.780	34.094	241.514
*P value*	< 0.001*	< 0.001*	< 0.001*	< 0.001*	< 0.001*

*P < 0.05

## Discussion

This study indicated that the ART knowledge scale was reliable in measuring the level of ART knowledge. Its four-factor solution can be used to measure ART knowledge that cover the correct use of ART, adherence, benefits, and side effects. Psychometric assessment documents strong internal consistency and adequate discriminant construct validity. In addition, the variation in the scale scores of different socio-economic characteristics indicated its usefulness to determine key populations who need to receive a knowledge-based intervention, as the initial step, in the promotion of ART to reduce disease progression and curb further HIV transmission.

It is generally accepted that a sample size of at least 10–20 times of the variables was required in scales assessment of reliability and validity [26]. The sample size of this study met the requirement as there were 14 items in the scale and the sample size was 410 participants. The Cronbach’s alpha for each dimension was adequate (0.70–0.95). The correlation coefficient between the four dimensions were statistically significant in the scale (0.35–0.62). A larger Cronbach’s alpha coefficient indicates higher internal consistency and more reliable scores measured by the scale [16].

Both exploratory and confirmatory factor analyses were conducted to assess the ART knowledge scale. The exploratory factor analyses helped to retain the factor structure for underlying set of variables hypothesized to measure the awareness of ART. Exploratory factor analyses found KMO values close to 1 and Bartlett’s spherical tested *P* < 0.001, indicating that the bias correlation was sufficiently small [27]. A total of four factors were extracted and their cumulative proportions were above the minimum criterion of 40% for the structural validity test [28]. The confirmatory factor analysis validated the hypothesized factor structure by the goodness-of-fit analysis. The goodness of fit indices indicated that the four-factor solution fit the data quite well [20, 29]. The scale is applicable for assess knowledge of ART among HIV case before and after interventions such as ART and health education. It can be used as a tool to evaluate the effectiveness of interventions.

Knowledge scores of ART differed among PLHs with different social economic characteristics. Knowledge scores were also associated with ART status and CD4 levels. As reported by other researchers [15, 30], PLHs with high ART knowledge scores were more likely to initiate ART and recover to normal CD4 levels. In addition, the cases with better knowledge were desire to experience the benefits of therapy and excellent adherence, which contributed to the outcomes [14, 30]. On the other hand, PLHs might acquire more ART knowledge after ART initiation [15, 31]. People with higher education level, none famers or migrant workers, might had more access to HIV-related knowledge [32]. PLHs with spouses/partners might had more motivation to grasp ART knowledge under the support of the partner or other family member [33, 34]. Different characteristics of PLHs should be taken into consideration on developing and implementing ART knowledge education programs.

## Limitation

Some limitations should be noticed. Because this study was conducted in one area (Honghe Prefecture), the findings may not be generalized to other areas. Future studies with larger sample sizes are needed to verify the proposed measurement model for the scale in independent samples. Since the HIV transmission routes in this study were sexual encounter, the scale might not be applicable to PLHs who contract HIV through other transmission routes.

## Conclusion

The scale indicated good internal consistency and adequate discriminant construct validity, and can be used to accurately measure the level of ART knowledge in intervention programs and assessment of ART use. PLHs with different social economic characteristics and HIV infection should be considered in the development and implementation of ART intervention programs that promote the use of ART and adherence.

## Data Availability

The datasets used and analyzed during the current study are available from the corresponding author on reasonable request.
